# Genomic landscape of colitis-associated cancer indicates the impact of chronic inflammation and its stratification by mutations in the Wnt signaling

**DOI:** 10.18632/oncotarget.22867

**Published:** 2017-12-12

**Authors:** Masashi Fujita, Nagahide Matsubara, Ikuo Matsuda, Kazuhiro Maejima, Ayako Oosawa, Tomoki Yamano, Akihiro Fujimoto, Mayuko Furuta, Kaoru Nakano, Aya Oku-Sasaki, Hiroko Tanaka, Yuichi Shiraishi, Raúl Nicolás Mateos, Kenta Nakai, Satoru Miyano, Naohiro Tomita, Seiichi Hirota, Hiroki Ikeuchi, Hidewaki Nakagawa

**Affiliations:** ^1^ Laboratory for Genome Sequencing Analysis, RIKEN Center for Integrative Medical Sciences, Tokyo, Japan; ^2^ Division of Lower Gastrointestinal Surgery, Department of Surgery, Hyogo College of Medicine, Nishinomiya, Japan; ^3^ Department of Surgical Pathology, Hyogo College of Medicine, Nishinomiya, Japan; ^4^ Department of Drug Discovery Medicine, Graduate School of Medicine, Kyoto University, Kyoto, Japan; ^5^ Laboratory of DNA Information Analysis, Human Genome Center, Institute of Medical Science, The University of Tokyo, Tokyo, Japan; ^6^ Department of Computational Biology and Medical Sciences, Graduate School of Frontier Sciences, The University of Tokyo, Kashiwa-shi, Chiba, Japan; ^7^ Laboratory of Functional Analysis in silico, Human Genome Center, The Institute of Medical Science, The University of Tokyo, Minato-ku, Tokyo, Japan; ^8^ Department of Inflammatory Bowel Disease Surgery, Hyogo College of Medicine, Nishinomiya, Japan

**Keywords:** inflammatory bowel disease, colitis-associated cancer, next-generation sequencing, RNF43, APC

## Abstract

Inflammatory bowel disease (IBD) increases the risk of colorectal cancer, known as colitis-associated cancer (CAC). It is still unclear what driver mutations are caused by chronic inflammation and lead to CAC development. To get insight into this issue, we investigated somatic alterations in CAC. We performed exome sequencing of 22 fresh CACs and targeted sequencing of 43 genes on 90 archive specimens from Japanese CAC patients, of which 58 were ulcerative colitis (UC) and 32 were Crohn’s disease (CD). Consistently with the previous reports, *TP53* was commonly mutated (66%) whereas *APC*, *KRAS* and *SMAD4* were mutated less frequently (16%, 11% and 11%, respectively). Mucinous CD-CACs in the anus, an Asian-specific subtype of CD-CAC, had less somatic mutations in our target genes. We also found that *RNF43*, a negative regulator of the Wnt signaling, was somatically mutated in a significant fraction of CACs (10 of 90; 11%). Two lines of evidence indicated that somatic mutations of *RNF43* were related to chronic inflammation. First, somatic mutations of *RNF43* were significantly associated with longer duration of IBD. Second, clinico-pathological features suggested many of the *APC*-mutated CACs were actually sporadic colorectal cancer whereas *RNF43*-mutated CACs did not have this tendency. RNA-Seq analysis showed that *RNF43*-mutated CACs had elevated expression of *c-Myc* and its target genes, suggesting that *RNF43* is a *bona fide* driver of CAC development. This study provides evidence that somatic mutation of *RNF43* is the driver genetic alteration that links chronic inflammation and cancer development in about 10% of CACs.

## INTRODUCTION

Ulcerative colitis (UC) and Crohn’s disease (CD) are collectively termed inflammatory bowel disease (IBD). In patients with IBD, long-term exposure to chronic inflammation is the primary risk factor for colorectal cancer development as analogous to liver cancer in chronic hepatitis and gastric cancer in chronic atrophic gastritis [1–3]. Malignancies in colorectum, anus, small intestine, or intestinal fistula which developed in IBD are described as colitis-associated cancer (CAC) or colitic cancer. In a meta-analysis, quantitative estimates of CAC risk in UC patients have been reported to be 2% after 10 years, 8% after 20 years, and 18% after 30 years of disease [4] and CAC risk was highly concordant with the location and extent of UC disease, with an incidence ratio of 1.7 for proctitis, 2.8 for left-sided colitis, and 14.8 for pan-colitis [5], which supports the strong association between chronic inflammation and cancer development. Similarly, CD increases the cumulative risk of CAC to 2.9% for 10 years, 5.6% for 20 years, and 8.3% for 30 years of disease [6]. Now intensive colonoscopic surveillance is clinically recommended to screen for CAC or its precursor dysplasia in patients suffering from longstanding IBD every 1–2 years [7], and cohort studies have demonstrated improved survival in patients with IBD undergoing colonoscopy [8].

The causal and clinical relationship between chronic inflammation in IBD and CAC is well established, but the molecular understanding of how chronic inflammation in IBD leads to CAC development is still incomplete. Sporadic colorectal cancer (CRC) is much better understood in terms of the molecular mechanism as explained by the adenoma-carcinoma sequence [9]. In this model, mutation of *APC* initiates adenoma, and subsequent mutations of *KRAS* and *TP53* progressively lead to invasive carcinoma. However, this model for sporadic CRC has been considered not to be concordant with CAC development. Mutation of *APC* was reported to be much less frequent in CACs, whereas somatic mutation of *TP53* was more prevalent and observed even in premalignant lesions [10]. A current model for CAC development is proposed to be the inflammation-dysplasia-carcinoma sequence although somatic alterations that advance the sequence are largely unclear [11].

The innovation of DNA sequencing technologies enabled cataloging somatic alterations in cancer comprehensively as already demonstrated by several cancer genome projects [12–15]. Two recent studies reported genomic analyses of 31 and 47 CAC samples, respectively [16, 17]. These studies found several recurrent mutations, including prevalent somatic mutations of *TP53* and less frequent somatic mutations of *APC* and *KRAS*. However, the previous studies may have missed low-frequency driver mutations because of their limited sample size.

Here we performed targeted sequencing of total 90 CACs, following exome sequencing of 22 frozen fresh CACs. We found that *RNF43* was somatically mutated in 11% of the CACs and the mutations preferentially occurred in patients with longer duration of IBD. RNA-Seq analysis showed that *RNF43*-mutated CACs had elevated expression of c-Myc and its target genes. Our study indicates that somatic mutation of *RNF43* is the molecular link between chronic inflammation and cancer development in approximately 10% of CAC cases.

## RESULTS

### Somatic mutations of *RNF43* were recurrently found in CAC

In order to search for driver genes of CAC development, we employed a two-step approach. The first step was exome sequencing of 22 CACs and enumeration of recurrently mutated genes. As the second step, we performed targeted sequencing of these genes and analyzed their somatic mutations in 90 CACs.

As the first step of our search, we collected 22 frozen samples of this rare type tumors with UC, and we performed exome sequencing of their fresh frozen tumor and normal colon tissues. Because the frozen tissues had low tumor purity, we sequenced the tumor exomes more deeply than the typical study. The average depth in the target region was 144-fold for tumor and 69-fold for normal tissues (Supplementary Table 3). Somatic single-nucleotide variants (SNVs) and short insertion/deletions (INDELs) were called as differences of tumor exomes from normal exomes by using VarScan2 [18]. One sample (RK378) was hypermutated, having 428 SNVs and 367 INDELs (Supplementary Figure 1A). This sample had a somatic missense mutation in a DNA mismatch repair gene *MLH1* (p.Ser295Ile). The other 21 samples had 72.2 SNVs and 10.2 INDELs on average (Supplementary Table 3). Somatic SNVs and INDELs in the 22 CACs affected 2112 genes (median 78 genes per patient, Supplementary Table 4). Among them, 262 genes were recurrently mutated. The 30 most frequently mutated genes included known driver genes *TP53, APC, KRAS* and *SMAD4* (Supplementary Figure 1B). This result suggests that these recurrently mutated genes are involved in CAC development and worth extensive investigation.

**Figure 1:**
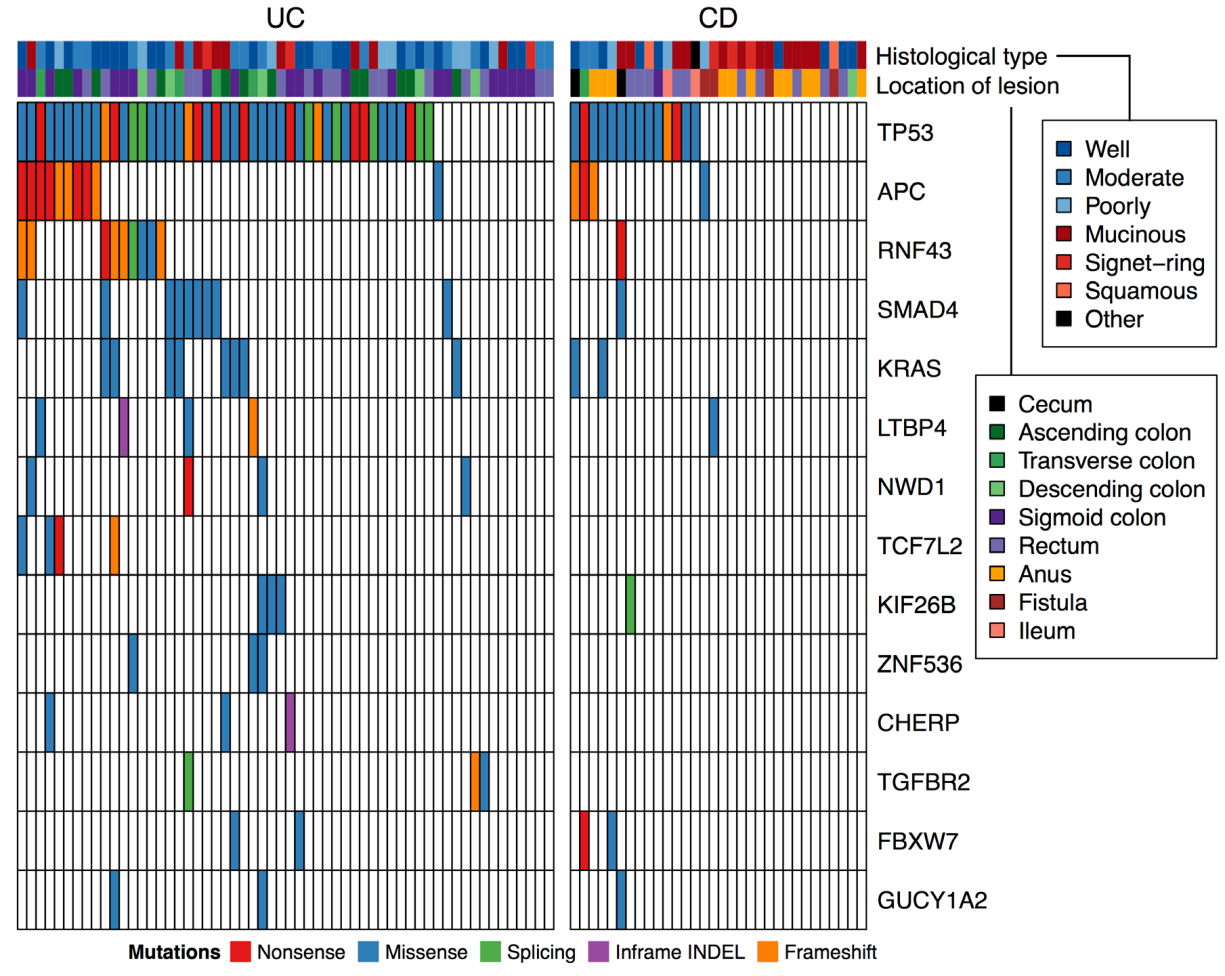
Recurrent somatic mutations in targeted sequencing of 90 CACs Genes mutated in 3 or more cases were shown.

As the second step of our search, we performed targeted sequencing of these recurrently mutated genes and analyzed their somatic mutations in a cohort of increased size. We collected FFPE specimen from 90 CAC tissues, of which 58 are associated with UC and 32 are associated with CD (Table 1) and dissected tumor cells to increase tumor purity for these FFPE samples. Fifteen of the 58 UC patients were identical to the patients analyzed by the exome sequencing. We selected 43 genes that had recurrent somatic mutations in exome analyses of this study and a previous study [16] as the target of sequencing. The targeted genes are listed in Supplementary Table 5. The average sequence depth in the target region was 496-fold (Supplementary Table 6). Somatic SNVs and INDELs were called by comparing tumor reads with the reference human genome and filtering common variants in the dbSNP database. All variants were examined by capillary sequencing of normal DNA, and the germline variants were filtered out. As a result, 144 somatic SNVs and 23 somatic INDELS were detected (Supplementary Table 7). The number of somatic variants per sample range from 0 to 8 (mean 1.86), and at least one somatic variant was detected in 69 of 90 samples. The somatic variants were found in 33 of 43 target genes. The most commonly mutated gene was *TP53*, affecting 59 of 90 patients (66%, Figure 1). Other frequently mutated genes were *APC, KRAS, SMAD4*, *RNF43,* and *LTBP4* (affecting 14, 10, 10, 10 and 5 patients, respectively). We examined the 33 genes that were somatically mutated in the 90 CACs for their association with tumor stages using the Wilcoxon rank sum test. However, no gene had statistically significant association with tumor stages after the Benjamini-Hochberg correction for multiple hypothesis testing.

**Table 1:** Summary of clinical features

Underlying disease		
	UC	58 (64%)
	CD	32 (36%)
Sex		
	Male	56 (62%)
	Female	34 (38%)
Age at cancer diagnosis		
	Median (range)	49 (27–79)
Age at IBD onset		
	Median (range)	28 (11–67)
Duration of IBD		
	Median (range)	17.8 yrs (0.2–40.5)
Stage		
	0	4 (4%)
	I	13 (14%)
	II	36 (40%)
	III	26 (29%)
	IV	6 (7%)
	NA	5 (6%)

Interestingly, somatic mutations of *RNF43* were frequently observed (10 of 90 patients; 11%). RNF43 negatively regulates the Wnt signaling pathway through its E3 ubiquitin ligase activity against frizzled receptors [19]. Frequent mutations of *RNF43* has been reported in sporadic CRC, endometrial cancer, gastric adenocarcinoma, and intraductal papillary mucinous neoplasms of the pancreas [13, 20, 21]. *LTBP4* (latent transforming growth factor beta binding protein 4) is an extracellular protein that regulates TGF-β bioavailability [22]. Mutations in *LTBP4* are common in recurrent glioblastoma, and silencing of LTBP4 impairs the proliferation of glioma cell lines [23]. To examine the function of LTBP4 in colorectal cancer, we knocked down *LTBP4* expression in a colon cancer cell line SW480 by siRNA and performed an invasion assay. Silencing of *LTBP4* promoted invasion and migration of the cells (Supplementary Figure 2), suggesting that LTBP4 plays a role also in colorectal cancer.

### Comparison of somatic mutations between CAC and sporadic CRC

**Figure 2:**
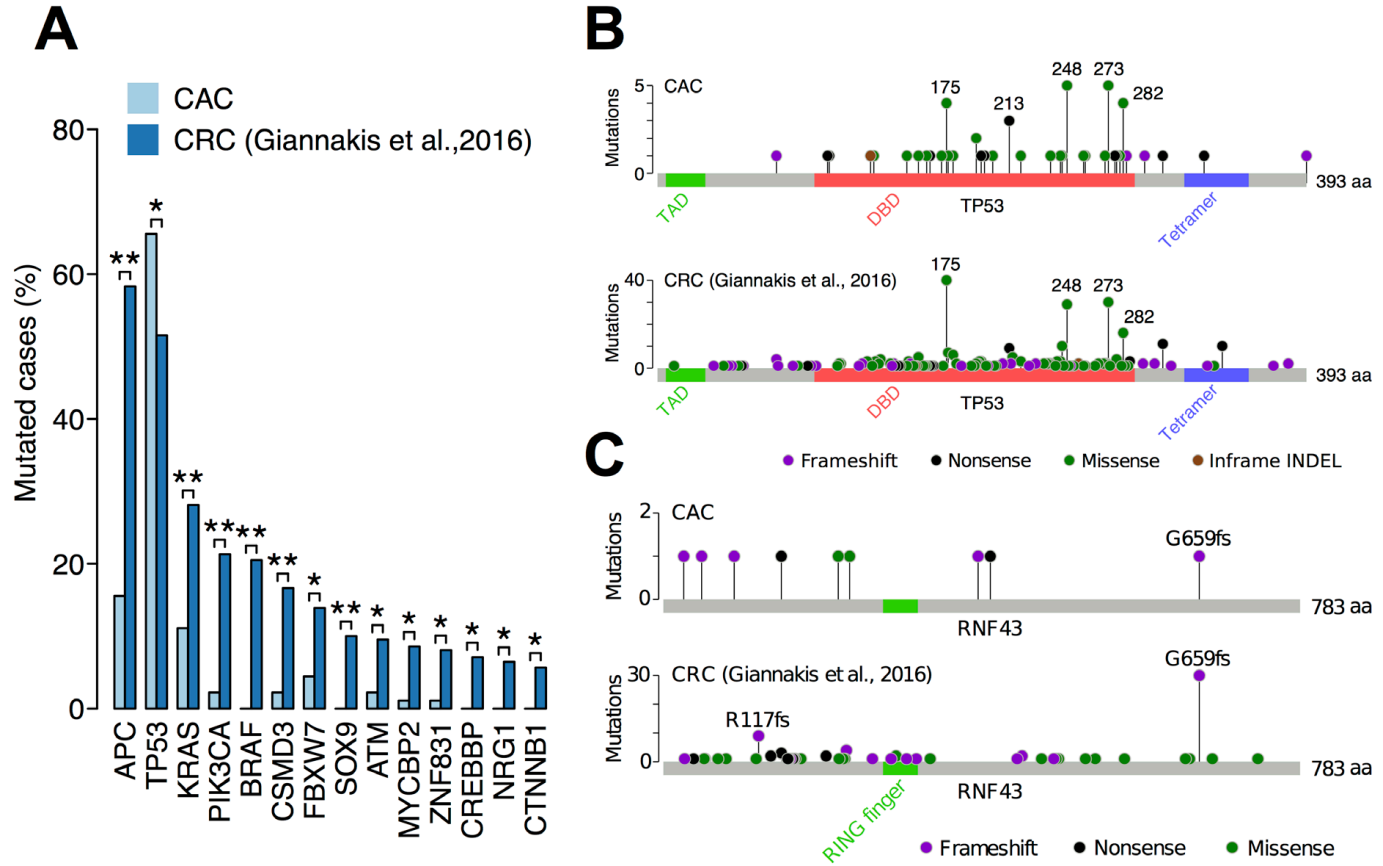
Comparison of somatic mutations between CAC and sporadic CRC (**A**) The frequency of somatic mutations in 90 CACs and 619 sporadic CRCs. Only genes that were captured in both studies and had a significant difference between them were shown. ^**^q-value < 0.01; ^*^q-value < 0.05 using Fisher’s exact test and the Benjamini-Hochberg procedure. (**B**) Distribution of somatic *TP53* mutations in CAC and sporadic CRC. TAD, transactivation domain; DBD, DNA-binding domain; Tetramer, tetramerization domain. (**C**) Distribution of somatic *RNF43* mutations in CAC and sporadic CRC.

We compared the frequency of somatic mutations between CAC and sporadic CRC (Figure 2A). *APC* had the largest difference, whose somatic mutation was found in 58% of sporadic CRCs but only in 16% of CACs. Other commonly mutated genes in sporadic CRCs, including *KRAS, PIK3CA* and *BRAF*, were less mutated in CACs. The only exception was *TP53*, which was more mutated in CAC (66%) than sporadic CRC (52%). Most of the somatic mutations of *TP53* were missense mutations (38 of 60 mutations) and occurred in the DNA-binding domain, especially at the hotspot codons 175, 248, 273 and 282 (Figure 2B). A previous study reported that mutation distribution of *TP53* was different between CAC and sporadic CRC [16]. We compared the types and distribution of *TP53* mutations between CAC and sporadic CRC but were not able to find a significant difference between them.

In contrast, distribution of somatic *RNF43* mutations was considerably different between CAC and sporadic CRC (Figure 2C). A large fraction of *RNF43* mutations in sporadic CRCs was frameshift INDELs at codons 659 and 117 (41 of 90 mutations). In contrast, somatic mutations of *RNF43* in CAC distributed differently, having only one mutation at the codon 659. This indicated that mutation mechanism of *RNF43* in CAC is different from that in sporadic CRC. A previous study found that most *RNF43* mutations in sporadic CRCs occur in cancers with high microsatellite instability (MSI) [20]. Therefore, somatic mutations of *RNF43* in CAC may arose from a mechanism different from MSI.

### Comparison of somatic mutations between UC and CD

Somatic mutations of our target genes were more frequent in UC-CAC than CD-CAC (Figure 1). *TP53* was mutated in 14 of 32 CDs (44%) and 45 of 58 UCs (78%; *p* < 0.01 by Fisher’s exact test). Likewise, samples with no somatic mutation among the 43 target genes were significantly more frequent in CD (16 of 32; 50%) than in UC (5 of 58; 9%; *p* < 10^−4^ by Fisher’s exact test). Because the target genes were selected based on the exomes of UC-CAC, we were not able to conclude that the mutation burden was lower in CD-CAC compared to UC-CAC. However, these mutations could be used for stratification of CD-CACs.

Actually, we noticed that somatic mutations in our target genes was inversely associated with a specific subtype of CD-CAC. Asian patients with CD have a unique tendency to develop mucinous adenocarcinoma in anal canal and hemorrhoid fistula [24, 25]. Histology of such CD-CACs in this study was shown in Supplementary Figure 3A. We noticed that mucinous CD-CACs in the anus were less likely to have somatic mutations in our target genes (Figure 1). Among the 16 CD-CACs without somatic mutation in our target genes, 6 cases (38%) were mucinous carcinomas developed in the anus. In contrast, among the 16 CD-CACs with somatic mutations in our target genes, none was mucinous carcinomas in the anus (0%, *p* < 0.05 by Fisher’s exact test). To search for somatic driver mutations of the 6 mucinous CD-CACs in the anus, we performed their exome sequencing. We also exome-sequenced 4 other CD-CACs without somatic mutation in our target genes (2 signet ring carcinomas in the anus and 2 mucinous carcinomas in the rectum). To increase the tumor cell fraction, we dissected cancer cells from FFPE tissues before DNA extraction. A search for recurrently mutated genes showed that fourteen genes (*ATRNL1, EPB41L3, GLI3, LOXL3, MROH2B, MUC3A, OTUD7B, RHOBTB1, RUNX2, SLC47A2, SLC6A3, TYMP, UNC13A,* and *ZDBF2*) were somatically mutated in 2 of 10 samples. But well-known cancer genes rarely had somatic mutations except one *TP53* mutation of low variant allele frequency and one *RB1* mutation. We also analyzed somatic copy number alteration but no recurrent alteration was identified. Therefore, driver mutations of the mucinous CD-CACs in the anus remained largely unclear.

A prognostic analysis showed that patients with CD had significantly poor overall survival than patients with UC (Supplementary Figure 3B). A multivariate analysis using Cox proportional hazards model showed that overall survival in this cohort was largely determined by tumor stage (hazard ratio 2.5, 95% CI 1.7–3.8) and underlying disease (UC or CD; hazard ratio 5.9, 95% CI 2.2–16.0).

### Somatic mutation of *RNF43* was associated with chronic inflammation

**Figure 3:**
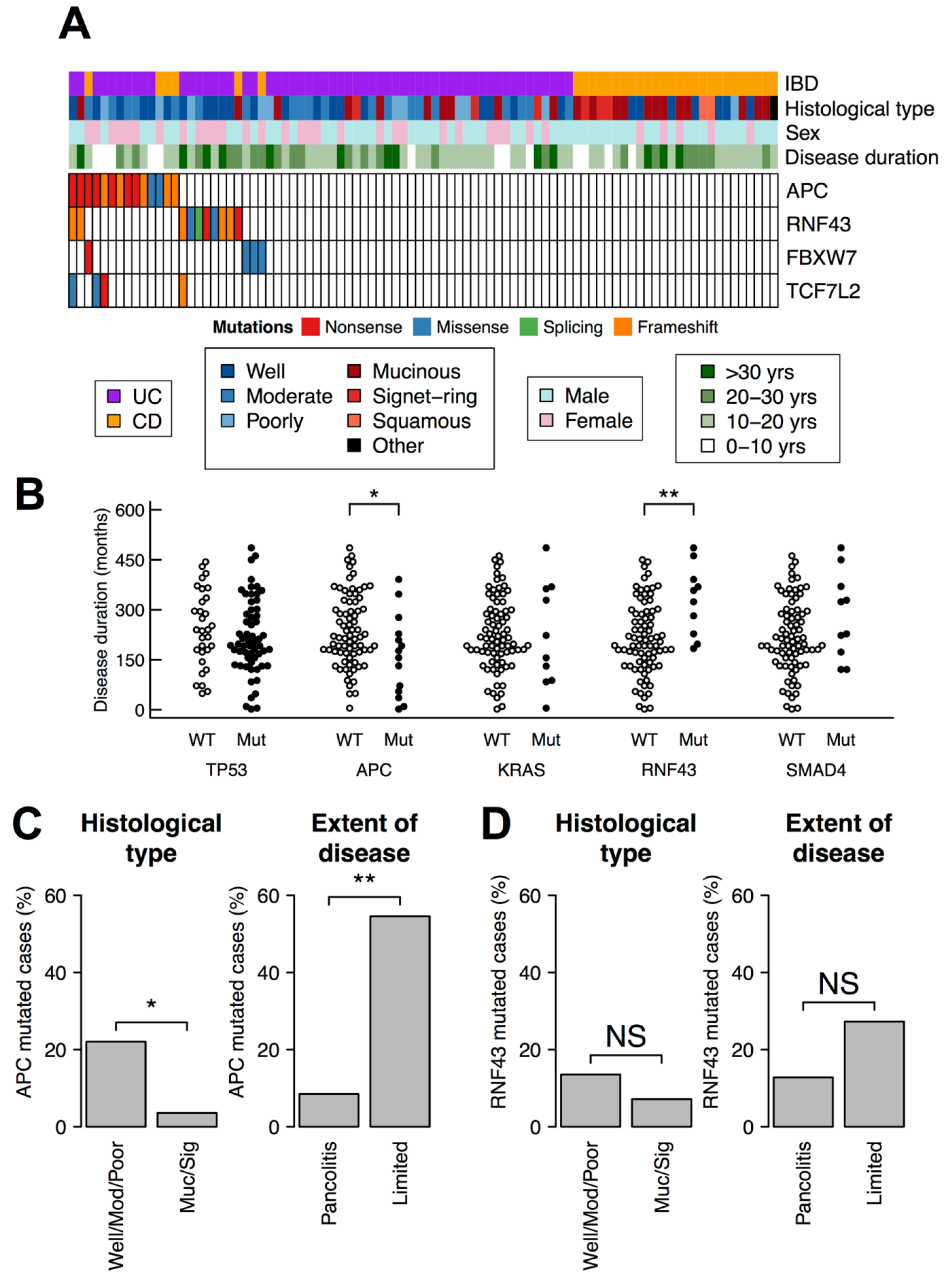
Somatic mutations of *APC* and *RNF43* and clinico-pathological features (**A**) Somatic mutations of *APC*, *RNF43*, and other two genes related to the Wnt pathway. (**B**) Somatic mutations and the duration of IBD. The statistical test was performed using Wilcoxon rank sum test. (**C**, **D**) Association of somatic mutations with histological type and the extent of disease. The extent of disease was available only in 58 UC cases. The statistical test was performed using Fisher’s exact test. (C) *APC* mutations. (D) *RNF43* mutations. ^**^p-value < 0.01; ^*^p-value < 0.05.

To infer driver mutations that arose from chronic inflammation, we compared somatic mutations and clinico-pathological features of our CACs (Figure 3A). The duration of IBD is an index of chronic inflammation and one of the highest risk factor for CAC development [4]. We found that *RNF43*-mutated patients had longer duration of IBD than non-mutated patients (mean 328 months and 215 months, respectively; *p* < 0.01 by Wilcoxon rank sum test) (Figure 3B). The duration rather than age was relevant because somatic mutations of *RNF43* did not associate with neither age at IBD onset nor age at cancer diagnosis (Supplementary Figure 4). This result raises the possibility that chronic inflammation is related with the somatic mutations of *RNF43*.

We also observed an inverse correlation between somatic mutations of *APC* and the duration of IBD. *APC*-mutated patients had shorter duration compared to non-mutated patients (mean 163 months and 239 months, respectively; *p* < 0.05 by Wilcoxon rank sum test) (Figure 3B), indicating that somatic mutations of *APC* were less related to chronic inflammation. Sporadic CRC can develop in patients of IBD, and discriminating such sporadic CRC from CAC is occasionally difficult. Our samples in this study may also contain sporadic CRCs in part. Because *APC* is the most frequently mutated gene in sporadic CRC, the inverse correlation suggests that many, if not all, of the *APC*-mutated CACs were actually sporadic CRCs.

This notion was further supported by two other clinico-pathological features. Firstly, mucinous adenocarcinoma and signet-ring cell carcinoma are known to be more frequent in CAC than in sporadic CRC [26, 27]. Secondly, the extent of IBD is reported to be a strong risk factor for CAC [5]. In our samples, *APC* was less frequently mutated in mucinous adenocarcinoma and signet-ring cell carcinoma (1 of 28; 4%) than in well, moderately, or poorly differentiated adenocarcinomas (13 of 59; 22%; *p* < 0.05 by Fisher’s exact test) (Figure 3C). Furthermore, *APC* was less frequently mutated in CACs originated from pancolitis (4 of 47; 9%) than in CAC with limited colitis (6 of 11; 55%; *p* < 0.01 by Fisher’s exact test).

Conversely, *RNF43* did not have these two trends (Figure 3D). These results suggest that, although both RNF43 and APC are negative regulators of the Wnt signaling, only somatic mutation of *RNF43* is associated with chronic inflammation of CACs.

### Somatic mutation of *RNF43* was associated with higher activity of c-Myc

**Figure 4:**
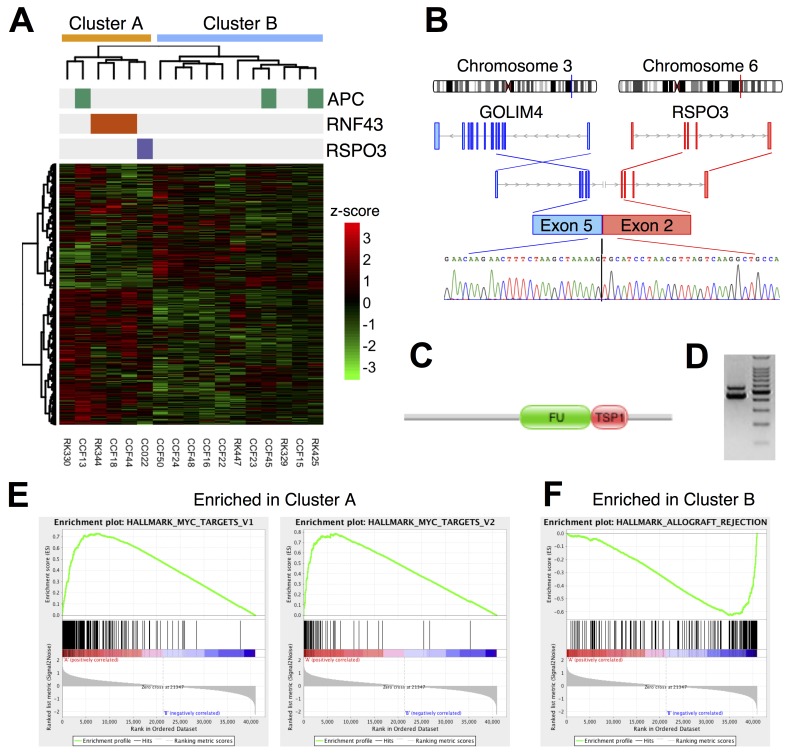
(**A**) Two-way clustering of transcriptome profiles in 17 UC-CAC samples with an annotation for somatic mutations. The clustering was performed by applying Ward’s method to log_10_(1+FPKM) values. (**B–D**) Gene fusion of *GOLIM4* and *RSPO3*. (B) A schematic gene structure. (C) Domain structure of the fusion protein. FU: Furin-like repeat; TSP1: Thrombospondin type-1 (TSP1) repeat. (D) RT-PCR of the junction site. A chromatogram of capillary sequencing for the lower band is shown at the bottom of (B). (**E, F**) Gene set enrichment analysis. (E) Two gene sets regulated by MYC were more expressed in the cluster A than the cluster B. (F) A gene set involved in allograft rejection were more expressed in the cluster B than the cluster A.

To examine whether somatic mutations of *RNF43* were associated with aberrant mRNA expression in CACs, we performed transcriptome profiling. Among the 22 fresh frozen UC-CACs, 17 tissues yielded high quality RNAs, and these RNAs were subjected to RNA-Seq analysis. A search for gene fusion using the RNA-Seq data identified a fusion between *GOLIM4* and *RSPO3* (R-Spondin 3) in one patient CC022 (Figure 4B–4D). RSPO3 activates the Wnt pathway by promoting membrane clearance of RNF43 and its functional homolog ZNRF3 [28]. Therefore, the *RSPO3* fusion may attenuate RNF43 and activate the Wnt pathway as reported in sporadic CRC [29].

Hierarchical clustering of the mRNA expression levels separated the 17 CACs into two clusters A and B, which consisted of 6 and 11 samples, respectively (Figure 4A). We found that the cluster A had significantly more somatic mutations in the Wnt pathway than the cluster B (5 of 6 in the cluster A; 2 of 11 in the cluster B; *p* < 0.05 by Fisher’s exact test). Three *RNF43* mutations belonged to the cluster A in conjunction with one *APC* mutation and the *RSPO3* fusion. The coherence of three *RNF43* mutations (and one *RSPO3* fusion) was intriguing because this suggested that these mutations affected common biological pathways in different patients.

To search for the biological pathways affected by the somatic mutations of *RNF43*, we analyzed differential gene expression between the clusters. The gene set enrichment analysis [30] showed that genes related to transplant rejection (including *CD4*, *CD8A*, *GZMA* and HLA class II) were highly expressed in the cluster B (*q*-value = 0.005; Figure 4F), suggesting intense infiltration of immune cells in the cluster B. Conversely, *MYC* and its target genes were expressed more in the cluster A than the cluster B (*q*-values < 0.05; Figure 4E). Considering the known regulatory relationship between RNF43, the Wnt/β-catenin signaling and c-Myc, the observed elevation of the c-Myc expression was consistently explained as a consequence of somatic mutations of *RNF43*, although contribution of many other mutations could not be ruled out. This result suggests that somatic mutations of *RNF43* functioned as driver events of CAC through the activation of the Wnt/β-catenin signaling and its downstream c-Myc signaling.

### Pattern of base substitutions in CAC

**Figure 5:**
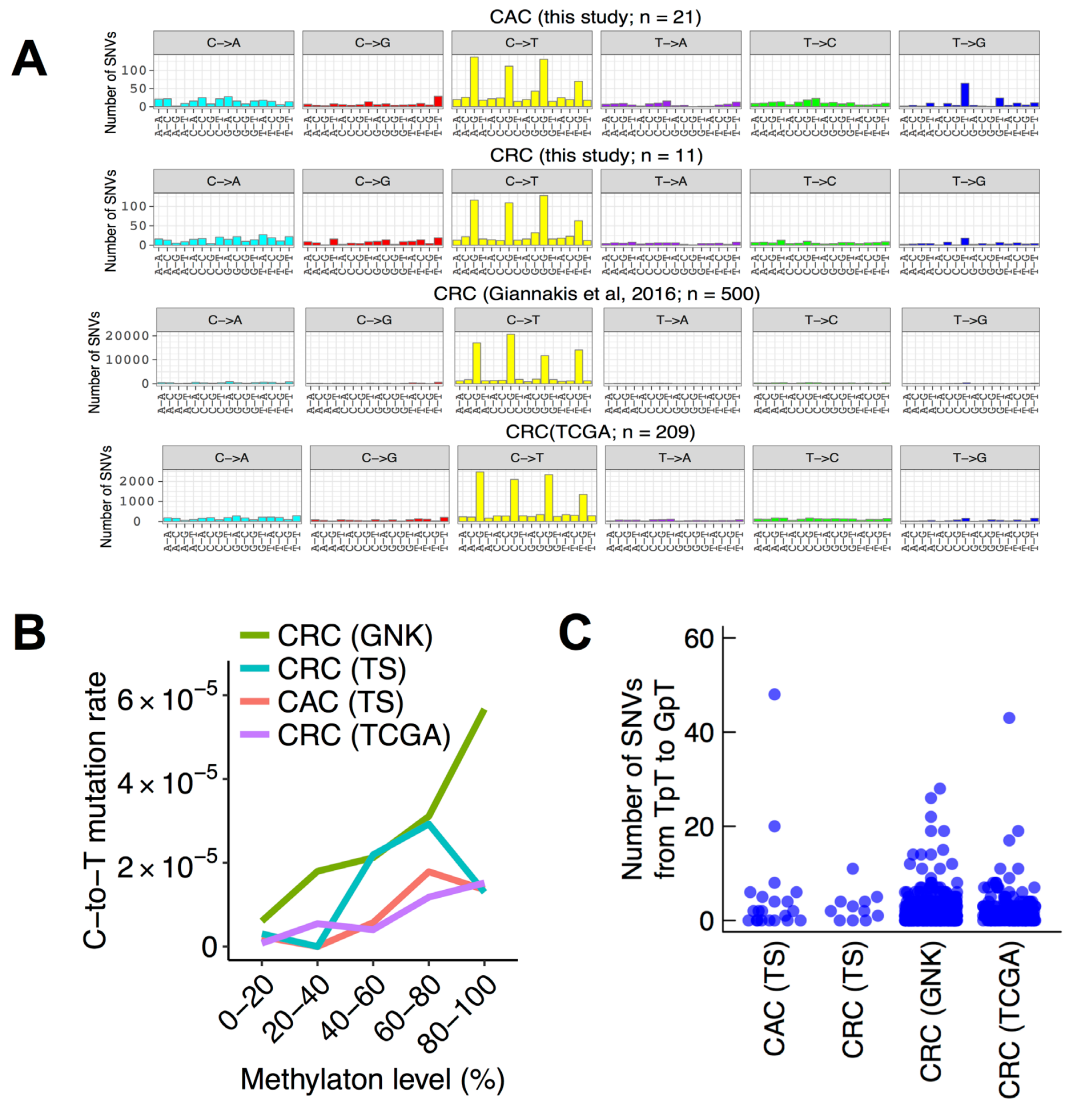
Trinucleotide context of base substitutions in CACs and sporadic CRCs (**A**) Number of trinucleotide substitution patterns in non-hypermutated colorectal cancers. Six possible substitutions from pyrimidine bases were further subdivided into 96 patterns based on the neighboring nucleotides. The four panels represent 21 CACs, 11 sporadic CRCs sequenced in this study, 500 sporadic CRCs in a previous study, and 209 sporadic CRCs in TCGA. (**B**) Methylation levels and somatic C-to-T mutation rate at CpG dinucleotides. Methylation levels were obtained from the ENCODE sigmoid colon data. (**C**) The number of SNVs from TpT to GpT in CAC and sporadic CRC. TS, this study; GNK, Giannakis *et al.*, 2016.

To gain insight into the mechanism of somatic mutation in CAC, we analyzed the patterns of base substitutions in the exome data of the fresh frozen tissues. Among the six possible patterns of base substitutions, the most frequent was C-to-T transitions, constituting 716 of 1517 SNVs (47%) in the 21 non-hypermutated CACs. We then analyzed the trinucleotide context of base substitutions. Among the 716 C-to-T transitions, 450 occurred in the CpG dinucleotide (63%, Figure 5A). It is commonly considered that C-to-T transitions in the CpG context is the result of spontaneous deamination of 5-methylcytosine. To corroborate this possibility, we compared somatic mutation rate at CpG dinucleotides with their methylation levels in the ENCODE sigmoid colon data. Methylation levels of CpG sites correlated positively with their C-to-T mutation rate in CAC (*p* < 0.001 by the Cochran–Armitage trend test; Figure 5B), further supporting a large contribution by 5-methylcytosine deamination to mutations in CAC. However, these observations were not specific to CAC. Three independent data sets of sporadic CRC also had similar trends (11 exomes sequenced in this study, 500 exomes published by Giannakis *et al.* [31], and 209 exomes by the TCGA project [12]) (Figure 5A, 5B).

A previous study reported that T-to-G transversions in the CpTpT context, which is a common mutation pattern in esophageal and gastric adenocarcinomas [13, 14], showed an excess in CAC but not in sporadic CRC [16]. Consistently with the previous observation, we found an excess of somatic SNVs from TpT to GpT in CAC but not in sporadic CRC (Figure 5A). Most of the 110 somatic SNVs from TpT to GpT were contributed by 2 of 21 CAC samples (48 and 20 SNVs by CC022 and RK447, respectively; Figure 5C). In contrast, sporadic CRC rarely had 20 or more somatic SNVs from TpT to GpT (this study, 0 of 11 samples; Giannakis *et al*, 3 of 500 samples; TCGA, 1 of 209 samples).

## DISCUSSION

In this study, we searched for recurrently mutated genes in 90 CACs employing a two-step approach. We first compiled a list of genes using the exome sequencing of 22 fresh frozen CACs and then performed targeted sequencing of 90 CAC archive specimens. We observed recurrent somatic mutations of *TP53, APC, KRAS* and *SMAD4*, consistently with previous reports [16, 17]. We also confirmed the previous findings that *TP53* was commonly mutated whereas the other three genes were less frequently mutated. This highlights the difference of CAC from sporadic CRC. In addition to these core genes, we also found that *RNF43* and *LTBP4* were somatically mutated in 11% and 6% of cases, respectively. A previous study of CAC also reported somatic mutations of *RNF43* in 6% of cases (3 of 47) [17].

Clinico-pathological analysis revealed that *APC*-mutated CACs were more likely to be sporadic CRCs. CAC tends to originate from pancolitis and more likely to show mucinous or signet-ring cell phenotypes compared to sporadic CRC [26, 27, 32]. However, somatic mutations of *APC* negatively associated with these features (Figure 3C). Moreover, *APC*-mutated CACs had significantly shorter duration of IBD than *APC*-wildtypes (Figure 3B). These data indicate that many of the *APC*-mutated CACs were sporadic CRC arose in the IBD background, although all the evidence is relative not absolute and there is always chance that these are indeed CACs.

In contrast to *APC*-mutated CACs, *RNF43*-mutated CACs did not resemble sporadic CRC. Somatic mutations of *RNF43* did not show bias toward sporadic CRC (Figure 3D). The distribution of mutated sites was also different between CAC and sporadic CRC (Figure 2C). More importantly, somatic mutations of *RNF43* correlated with the duration of IBD, indicating a relationship between chronic inflammation and the mutations (Figure 3B). RNA-Seq analysis showed that *RNF43*-mutated CACs belonged to a distinct subtype, in which target genes of c-Myc were upregulated. Considering these results, we propose that *RNF43* is the driver gene of CAC development in about 10% of cases.

The risk of CAC increases over the duration of IBD, but the molecular mechanism behind this phenomenon has been unclear. A likely explanation is that chronic inflammation somehow causes somatic alterations of genome, and some of the alterations subsequently drive CAC development. Therefore, which gene is altered by chronic inflammation and drives CAC is an important question for understanding CAC. An attractive candidate would be the gene that is frequently mutated only after the long duration of IBD, and *RNF43* appeared to have this association (Figure 3B). Therefore, we propose a hypothesis that chronic inflammation of IBD somatically mutates *RNF43* and the mutation drives CAC development, thereby increasing the CAC risk over disease duration. However, we have to admit that our hypothesis has two downsides. First, this hypothesis accounts for only 10% of CAC cases. Other mechanisms may operate on the remaining cases. Secondly, the association between mutations and disease duration alone is not conclusive evidence that somatic mutation of *RNF43* is sufficient to drive CAC development. Further studies of *RNF43* mutations, especially in premalignant mucosa, will be required to clarify the relationship between chronic inflammation, somatic mutation of *RNF43*, and CAC development.

*LTBP4* was the another recurrently mutated gene in this study. To the best of our knowledge, somatic mutations of *LTBP4* has not been linked to colon cancer. In particular, we confirmed that silencing *LTBP4* promotes invasion of a colon cancer cell line. In conjunction with *LTBP4*, genes in the TGFβ pathway (*SMAD4, SMAD2, TGFBR2, ACVR1B* and *LTBP4*) were somatically mutated in 19 of 90 patients (21%), suggesting that this pathway also contributes significantly to CAC development.

This study could not detect any recurrent driver mutation in a half of CD-CACs. In Japan, common type of CD-CACs are mucinous carcinoma in anal canal and hemorrhoid fistula [24, 25] and unknown genomic alterations could affect this distinct CD-CAC phenotype specific in Asia. We found that 14 genes were recurrently mutated in these CD-CACs, but unknown other genetic or epigenetic events could drive the carcinogenesis in this type of CACs in Asian CD disease. Overall, genetic events of CAC appear to have heterogeneity depending on the background disease, histology and ethnicity.

The patterns of base substitutions provide an insight into mutation mechanisms in cancer. A previous study analyzed FFPE samples of CAC and showed that base substitutions in CAC were dominated by C-to-T transitions at the CpG dinucleotides, indicating the effect of spontaneous deamination of 5-methylcytosine [16]. The authors also reported a slight excess of SNVs from TpT to GpT, resembling esophageal and gastric adenocarcinomas. We analyzed the patterns of base substitutions in the exome sequences of 21 fresh frozen non-hypermutated CACs, which had no bias of FFPE, and obtained essentially the same result to the previous study. However, the substitution patterns in CAC were largely similar to that of sporadic CRC. Although the excess of SNVs from TpT to GpT was specific to CAC, the excess was contributed by only a part of samples (2 of 21). We concluded that the mutation patterns of most CAC samples were identical to that of sporadic CRC.

This study provides a detailed profile of somatic mutations in 90 CACs. The profile revealed that *RNF43* was mutated and functioned as a cancer driver in about 10% of cases. The profile also confirmed that somatic mutations of *APC* could be informative for discriminating CAC from sporadic CRC in patients with IBD. These findings may have diagnostic and therapeutic utility for the treatment of CAC.

## MATERIALS AND METHODS

### Clinical samples

Fresh-frozen cancer tissues and normal tissues from UC patients (*n* = 22) and sporadic colorectal cancers without IBD background (*n* = 11) in Hyogo College of Medicine Hospital underwent exome and RNA-Seq. The Institutional Review Boards at RIKEN and Hyogo College of Medicine approved this work. All subjects agreed with informed consent to participate in the study following ICGC guidelines. Their clinico-pathological features are in Supplementary Table 1. DNA and RNA were extracted from fresh-frozen tumor specimens and adjacent normal tissues. FFPE (formalin-fixed paraffin-embedded) tissues (*n* = 90) of CACs in Hyogo College of Medicine Hospital were sliced, and after reviewing their HE-stained slides, tumor regions and normal regions were dissected and DNAs/RNAs were extracted by Qiagen Allprep kit.

### Whole exome and targeted sequencing

Exome capture was carried out using SureSelect XT Exome V5 Custom kit (Agilent Technologies). The exome-captured libraries were sequenced on HiSeq2000/2500 with paired reads of 100–125 bp. For DNAs from FFPE samples, we constructed sequencing libraries by using KAPA HyperPrep Plus kit. We selected 43 genes and performed target capturing by SureSelect XT Target Enrichment System. Targeted sequencing was performed on HiSeq2000/2500 with paired reads of 125 bp.

### Variant calling

Reads were adaptor-trimmed using Cutadapt [33] and mapped to GRCh37 using BWA [34]. PCR duplicates were removed using Picard (https://broadinstitute.github.io/picard/). Low-quality reads were filtered based on mapping quality, number of mismatches and INDELs. Improper reads were filtered based on discordance in chromosome, direction and distance of paired-end reads. For the exome data of freshly frozen samples, somatic single-nucleotide variants (SNVs) and short insertions/deletions (INDELs) were called using VarScan2 [18] with a minimum read depth of 20, a minimum variant allele frequency of 5%, minimum supporting reads of 4, and a *p*-value threshold of 0.05. For the targeted sequencing data of FFPE samples, somatic variants were called as follows. The numbers of matches, mismatches and INDELs at each chromosomal position were counted while skipping bases of quality <20 and alignments of mapping quality <10 using SAMtools (version 1.3.1). Common variants in dbSNP version 138 were discarded. SNVs were called when mismatches had read depth >50, variant allele frequency >5%, base quality bias >0.01, mapping quality bias >0.01, and read position bias >0.01. INDELs were required to have read depth >50, variant allele frequency >10% and support reads >20. Variants were discarded if identical alleles were detected in more than 8 samples. Copy number analysis was performed using VarScan2 and DNAcopy.

### RNA-Seq

RNA-Seq was carried out for 17 frozen CAC samples for which high-quality RNA was available. Total RNA was extracted by Qiagen Allprep kit from the frozen CACs and quantity were evaluated by Bioanalyzer (Agilent). The high-quality RNA was subject to polyA+ selection and chemical fragmentation, and 100–200 base RNA fraction was used to construct cDNA libraries according to Illumina’s protocol. RNA-Seq was performed on HiSeq2500 using the standard paired-end 125 bp protocol. RNA-Seq reads were adaptor-trimmed using Cutadapt [33] and mapped to GRCh37 using STAR [35]. Reads mapped onto exons were counted using featureCounts [36] and converted into FPKM-UQ. For biclustering of samples and genes, the FPKM-UQ values of the genes expressed in all of the 17 samples were log-transformed with pseudocount 1.0, scaled for each gene, and clustered using the Euclidean distance and Ward’s method implemented in R. Gene fusion was detected using fusionfusion (https://github.com/Genomon-Project/fusionfusion).

### Knockdown experiments and invasion assays

A colon cancer cell line SW480 was used for knockdown experiments of a new target gene (*LTBT4*). The cell lines were obtained from the Japanese Collection of Research Bioresources Cell Bank (Osaka, Japan). Cells were seeded on 24-well plates a day before transfection (1 × 10^5^ cells/well for growth assay of *LTBP4*), and transfected with two pre-designed siRNAs or negative control SIC, respectively, using Lipofectamine RNAiMAX (Invitrogen, Carlsbad, CA, USA) according to the manufacturer’s instruction. Sequence information of siRNAs are listed in Supplementary Table 2. The knockdown efficiency was assessed by quantitative RT-PCR using primers: 5′-GTCTCCAACGAGAGCCAGAG-3′ and 5′-GGCAGCAGCACTCTGTGTAG-3′. For the cell proliferation assay, cells were seeded in 24-well plate format a day before transfection. The cell numbers in triplicate wells were assessed by water-soluble tetrazolium salt (WST) assay (Dojindo, Kumamoto Japan) 96 hours after siRNA transfection. Invasion assay was performed in 24-well modified Boyden chambers pre-coated with Matrigel (BD Transduction, Franklin Lakes, NJ, USA). Cells were transfected with siRNAs in 6-well plate format using Lipofectamine RNAiMAX according to the manufacturer’s instructions. A day after siRNA transfection, appropriate numbers of each cells were transferred into the upper chamber. Following 24 hours of incubation, the migrated or invasive cells on the lower surface of filters were fixed and stained with the Diff-Quik stain (Sysmex, Kobe, Japan), and stained cells were counted directly with three different field of microscopy. The numbers of viable cells in each condition were assessed by WST assay at the same time point and the numbers of cells on invasion or migration assay were normalized with the ratio of viability score in SIC transfectants. Differences between subgroups were tested by Student’s *t*-test and considered significant at the *p-*value < 0.05 level.

## SUPPLEMENTARY MATERIALS FIGURES AND TABLES














