# Supplementary material for: Genomic landscape of colitis-associated cancer indicates the impact of chronic inflammation and its stratification by mutations in the Wnt signaling
Source: Oncotarget. 2017 Dec 12;9(1):969–81. doi: 10.18632/oncotarget.22867 (PMC5787528; doi:10.18632/oncotarget.22867)
Supplement: Supplementary file 1 [file oncotarget-09-969-s001.pdf]

Genomic landscape of colitis-associated cancer indicates the impact of chronic inflammation and its stratification by mutations in the Wnt signaling

SUPPLEMENTARY MATERIALS

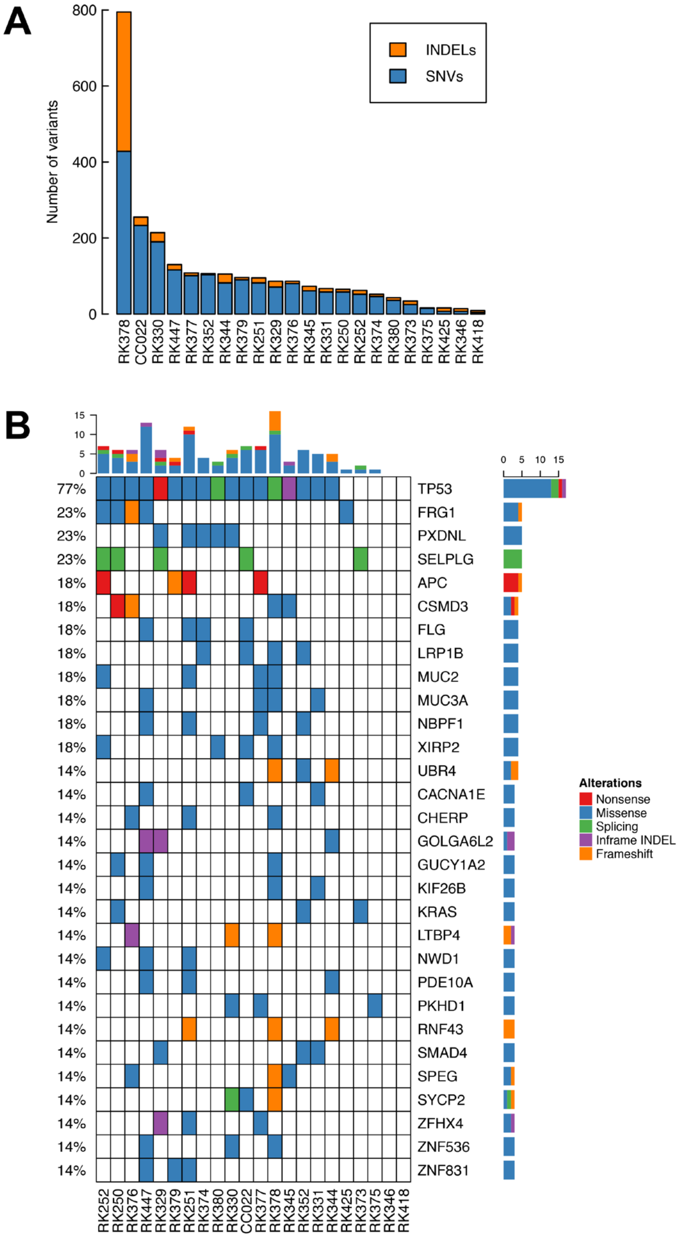

Supplementary Figure 1: Mutations in the whole-exome sequencing of 22 UC-associated cancers. (**A**) Number of somatic mutations. (**B**) Recurrently mutated genes. Genes that were mutated in more than 2 patients and had mutation rate of >10/Mbp were shown.

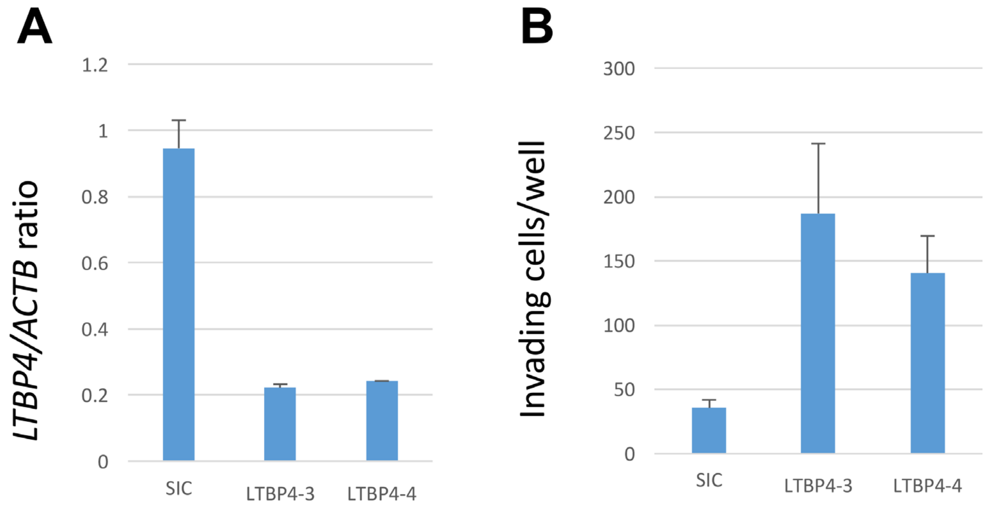

Supplementary Figure 2: Silencing *LTBP4* promoted invasion of SW480 colon cancer cell line. (**A**) Efficacy of LTBP4 silencing as measured by quantitative RT-PCR. (**B**) Invasiveness of LTBP4-silenced cells.

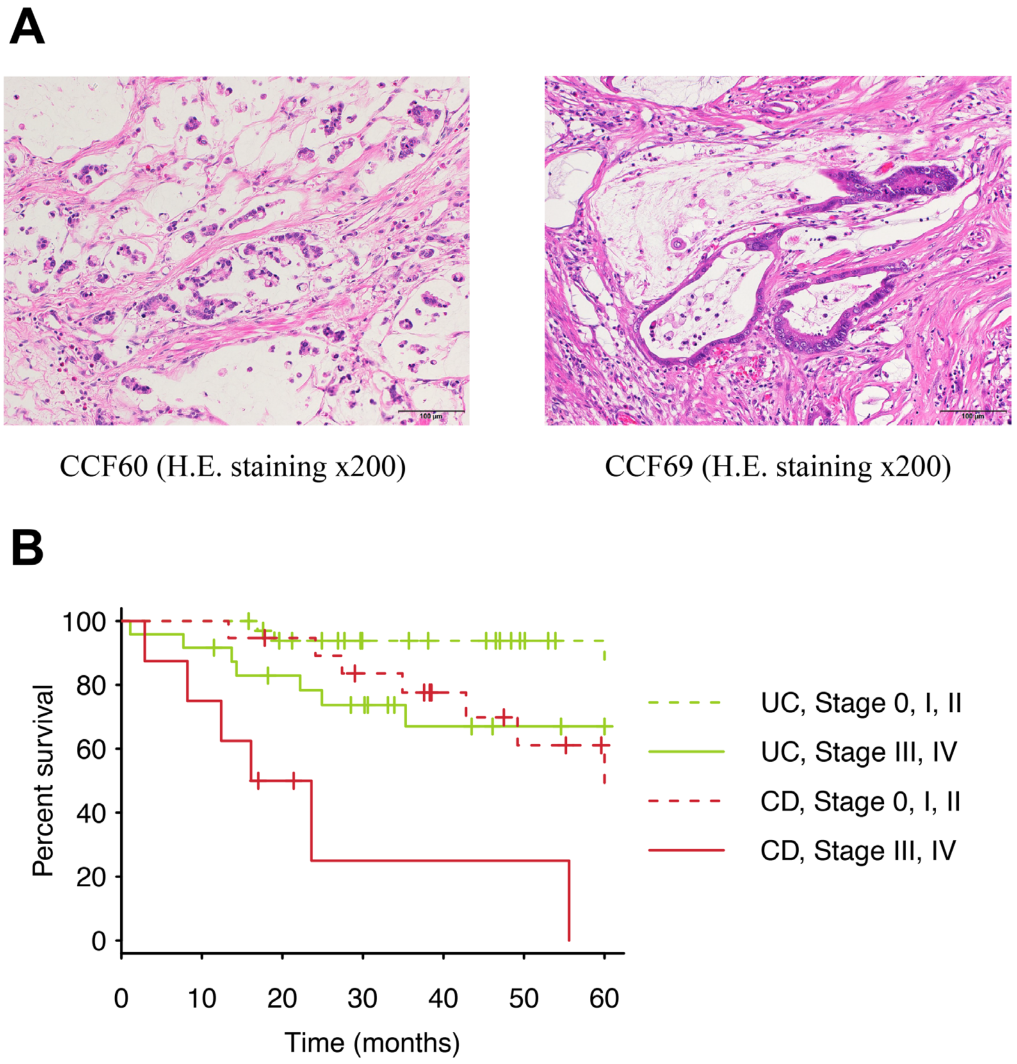

Supplementary Figure 3: Unique features of CD-CAC. (**A**) Mucinous carcinoma arising in anal canal and hemorrhoid fistula in Japanese Crohn's disease. CCF60 (H.E. staining ×200) and CCF69 (H.E. staining ×200). (**B**) Overall survival of CAC patients. Patients were grouped as follows: UC, stage 0, I, II ($n = 34$); UC, stage III, IV ($n = 24$); CD, stage 0, I, II ($n = 19$); CD, stage III, IV ($n = 8$).

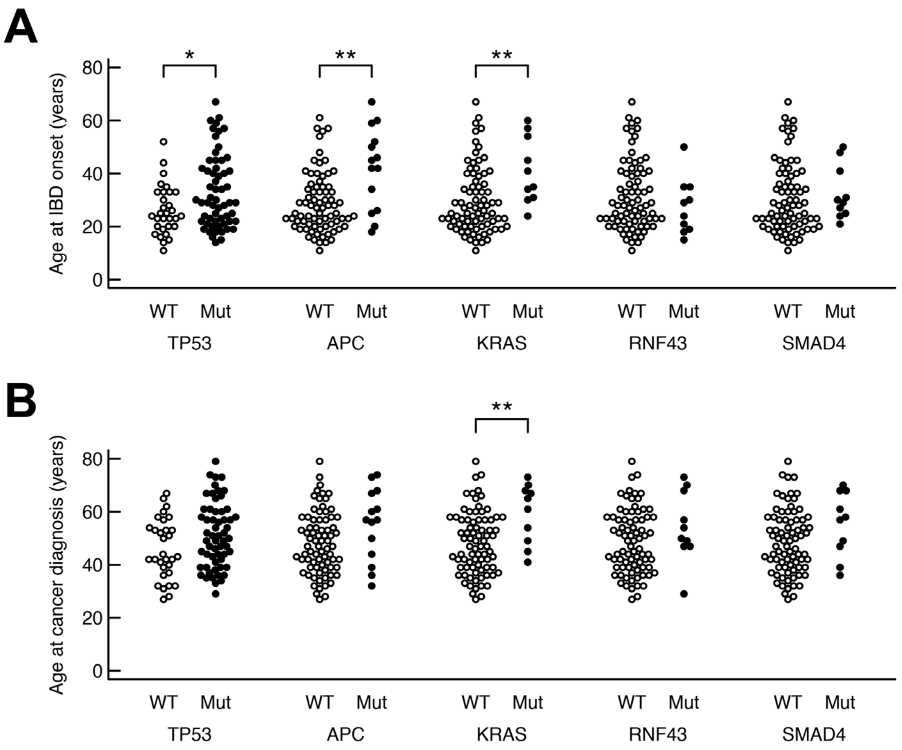

Supplementary Figure 4: Association of mutations and timeline of disease in 90 CACs. (A) Mutations and the age at IBD onset. (B) Mutations and the age at cancer diagnosis. $^{**}p$-value < 0.01; $^{*}p$-value < 0.05 using Wilcoxon rank sum test.

Supplementary Table 1: Clinico-pathological features of the colitis-associated cancers (CACs). See Supplementary_Table_1

Supplementary Table 2: Sequence information of siRNAs

Duplex name	Sense	Antisense
LTBP4-3	5′-CUCCUUUGCCUGUACUUGUdTdT-3′	5′-ACAAGUACAGGCAAAGGAGdTdT-3′
LTBP4-4	5′-CAGACUUAGGUCCACCUUAdTdT-3′	5′-UAAGGUGGACCUAAGUCUGdTdT-3′

Supplementary Table 3: Summary of exome sequencing of 22 CACs. See Supplementary_Table_3

Supplementary Table 4: Somatic mutations in the 22 CAC exomes. See Supplementary_Table_4

Supplementary Table 5: Targeted genes for resequencing of the 90 CAC. See Supplementary_Table_5

Supplementary Table 6: Sequencing metrics in the targeted resequencing of 90 CACs. See Supplementary_Table_6

Supplementary Table 7: Mutations in the targeted resequencing of 90 CACs. See Supplementary_Table_7
